# Correcting for Naturally Occurring Mass Isotopologue Abundances in Stable-Isotope Tracing Experiments with PolyMID

**DOI:** 10.3390/metabo11050310

**Published:** 2021-05-12

**Authors:** Heesoo Jeong, Yan Yu, Henrik J. Johansson, Frank C. Schroeder, Janne Lehtiö, Nathaniel M. Vacanti

**Affiliations:** 1 Division of Nutritional Sciences, Cornell University, Ithaca, NY 14853, USA; hj436@cornell.edu; 2 Boyce Thompson Institute and Department of Chemistry and Chemical Biology, Cornell University, Ithaca, NY 14853, USA; yy749@cornell.edu (Y.Y.); schroeder@cornell.edu (F.C.S.); 3 Science for Life Laboratory, Department of Oncology-Pathology, Karolinska Institutet, 17165 Solna, Sweden; henrik.johansson@ki.se (H.J.J.); janne.lehtio@ki.se (J.L.)

**Keywords:** stable-isotope tracing, mass isotopologue distribution, correction, metabolic flux analysis, natural abundances, metabolism

## Abstract

Stable-isotope tracing is a method to measure intracellular metabolic pathway utilization by feeding a cellular system a stable-isotope-labeled tracer nutrient. The power of the method to resolve differential pathway utilization is derived from the enrichment of metabolites in heavy isotopes that are synthesized from the tracer nutrient. However, the readout is complicated by the presence of naturally occurring heavy isotopes that are not derived from the tracer nutrient. Herein we present an algorithm, and a tool that applies it (PolyMID-Correct, part of the PolyMID software package), to computationally remove the influence of naturally occurring heavy isotopes. The algorithm is applicable to stable-isotope tracing data collected on low- and high- mass resolution mass spectrometers. PolyMID-Correct is open source and available under an MIT license.

## 1. Introduction

**Figure 1:**
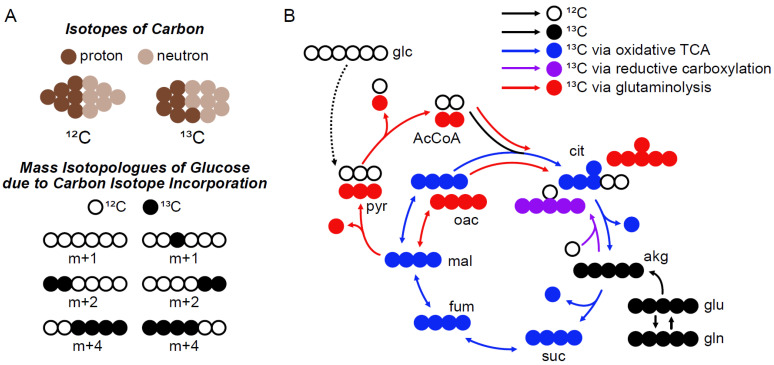
Principles of Stable-Isotope Tracing. (**A**) Isotopes of an element are atoms of that element with different masses due to the number of neutrons they contain. Each element has a characteristic isotope mass distribution (IMD) describing the proportion of the atoms having the nominal mass (m) and those greater than nominal mass by n integer atomic mass units (m + n). Mass isotopologues of a compound are molecules of that compound with different masses due to the number of heavy isotopes they contain. Each compound has a mass isotopologue distribution (MID) describing the proportion of molecules having the monoisotopic mass (m) and those greater than the monoisotopic mass by n integer atomic mass units (m + n). Molecules of mass m have no extra neutrons due to heavy isotope incorporation while those of mass m + n have n extra neutrons due to heavy isotope incorporation. (**B**) Schematic of possible TCA cycle metabolite labeling patterns due to incorporation of ^13^C from a [U-^13^C_5_]glutamine tracer.

Utilization of intracellular metabolic pathways can be assayed by tracing with stable-isotope-labeled nutrients. Cells cultured in medium containing a stable-isotope-labeled nutrient, i.e., a tracer, internalize and metabolize it; allowing the heavy isotope(s) to be incorporated in metabolic pathway reactants, products, and intermediates. Heavy isotope presence in metabolites can then be assayed by mass spectrometry and used to infer pathway utilization by quantitative modelling using metabolic flux analysis (MFA) or manual inspection of mass isotopologue distributions (MIDs, Figure 1A,B).

If MIDs are incorporated into an MFA model, they are input as either time-course or static (steady-state) measurements and the set of metabolic fluxes that minimizes the sum of squared differences between model-computed and measured values is returned. However, MFA models are generally constructed taking great liberties in making simplifying assumptions of biochemical networks, limiting their representation of cellular metabolism. Thus, manual interpretation is generally performed in lieu of the burdensome step of fitting an MFA model to measured MIDs.

As an illustration of flux inference by manual inspection, consider a glutamine tracer with all five carbons labeled as ^13^C, denoted [U-^13^C_5_]glutamine, that is metabolized by enzymes of the tricarboxylic acid (TCA) cycle and associated anaplerotic pathways. Examination of label incorporation on citrate can then distinguish relative utilization of the oxidative TCA cycle, reductive carboxylation, and glutaminolysis. If via the oxidative TCA cycle, [U-^13^C_5_]glutamine will label citrate with four heavy carbon atoms; if via the reductive carboxylation, [U-^13^C_5_]glutamine will label citrate with five heavy carbon atoms; if via glutaminolysis, [U-^13^C_5_]glutamine will label citrate with six heavy carbon atoms (Figure 1B).

However, inference of pathway utilization by manual inspection of metabolite MIDs is confounded by incorporation of naturally occurring heavy isotopes. Posed as a question related to the above described example using a [U-^13^C_5_]glutamine tracer: How does the researcher know if citrate with five heavy isotopes derives them all from the tracer or derives four from the tracer and one due to incorporation of naturally present ^13^C, ^2^H, or ^17^O? Whether synthesis of the citrate molecule in question is attributed to oxidative TCA cycle flux or reductive carboxylation is dependent on distinguishing these two scenarios (Figure 1B). Note that [U-^13^C_5_]glutamine labeling citrate with five heavy carbon atoms via simultaneous flux through glutaminolysis and pyruvate carboxylase is not considered in this example for simplicity.

This and analogous questions can be answered by post-processing of measured mass isotopologue distributions in a manner that removes the influence of naturally occurring heavy isotopes. Herein, we present the PolyMID-Correct tool, part of the PolyMID software package, that relies on known distributions of naturally occurring atomic isotopes [1] and applies the principles of polynomial expansion to correct stable-isotope tracing data for the presence of naturally occurring heavy isotopes.

PolyMID-Correct is open source and written in Python 3. Information about the metabolite, including its measured MID, is passed to this tool within a user-created text file or via the command line. The user can specify whether mass differences due to naturally occurring isotopes of specified atoms are resolved from mass differences due to the tracer atom, i.e., if the data is high mass resolution; or if MIDs are inclusive of all heavy isotopes, i.e., if the data is low mass resolution (henceforth referred to as high- and low- resolution). The identity of the tracer atom is also specified by the user along with the purity of the tracer molecule and the atom enrichment of the heavy-isotope label on the tracer.

Other programs that are freely available to correct for naturally occurring isotopes in high- and low- resolution data include AccuCor [2], IsoCor [3], IsoCorrectoR [4], and PyNAC [5]. However, PolyMID-Correct is the only of these that allows users to specify input parameters programmatically; allowing for smooth integration into data processing pipelines. PolyMID-Correct is also the only option that allows users to specify the elemental identity of atoms whose heavy isotope incorporation into metabolites is resolved from incorporation of heavy isotopes of the tracer element. AccuCor and IsoCor make this distinction based on the user-input instrument resolution whereas IsoCorrectoR and PyNAC handle high resolution data by considering all other elemental heavy isotopes to be distinguished from the tracer atoms. PolyMID-Correct also includes the option for data to be input from a single, simply formatted, text file; providing a shallower learning curve for command-line novices. PolyMID-Correct, IsoCor, and PyNAC are implemented in Python whereas AccuCor and IsoCorrectoR are implemented in R.

## 2. Results

### 2.1. General Correction Algorithm

PolyMID-Correct transforms a measured MID of a metabolite into a set of component MIDs. Each component MID is a theoretical MID of the metabolite where a known number of heavy tracer atoms are incorporated. Consider a [U-^13^C_6_]glucose tracing experiment where ^13^C incorporation on the amino acid serine is being examined. Theoretically, serine can be labeled with zero, one, two, or three heavy carbon atoms from [U-^13^C_6_]glucose. The portion of serine molecules containing zero ^13^C atoms derived from [U-^13^C_6_]glucose will have an MID consistent with naturally occurring isotope incorporation. The portion of serine containing one ^13^C atom derived from [U-^13^C_6_]glucose will also have an MID consistent with naturally occurring isotope incorporation with the exception that one of the carbon atoms is guaranteed to be labeled as ^13^C. Analogously, the portions of serine containing two or three ^13^C atom derived from [U-^13^C_6_]glucose will have MIDs consistent with naturally occurring isotope incorporation with the exceptions that, respectively, two or three of the carbon atoms are guaranteed to be labeled as ^13^C. The MIDs accounting for natural isotope incorporation of serine guaranteed to contain zero, one, two, or three ^13^C atoms derived from [U-^13^C_6_]glucose can be computed theoretically, as described in a subsequent section. Thus, the measured MID of serine can be formulated as Equation (1) where the weights of each of the component vectors are the components of the corrected MID.

(1) $$\left[\begin{array}{c} S_{m,M0}\\ S_{m,M1}\\ S_{m,M2}\\ S_{m,M3}\\ S_{m,M4}\\ S_{m,M5} \end{array}\right]=S_{c,M0}*\left[\begin{array}{c} S_{0L,M0}\\ S_{0L,M1}\\ S_{0L,M2}\\ S_{0L,M3}\\ S_{0L,M4}\\ S_{0L,M5} \end{array}\right]+S_{c,M1}*\left[\begin{array}{c} S_{1L,M0}\\ S_{1L,M1}\\ S_{1L,M2}\\ S_{1L,M3}\\ S_{1L,M4}\\ S_{1L,M5} \end{array}\right]+S_{c,M2}*\left[\begin{array}{c} S_{2L,M0}\\ S_{2L,M1}\\ S_{2L,M2}\\ S_{2L,M3}\\ S_{2L,M4}\\ S_{2L,M5} \end{array}\right]+S_{c,M3}*\left[\begin{array}{c} S_{3L,M0}\\ S_{3L,M1}\\ S_{3L,M2}\\ S_{3L,M3}\\ S_{3L,M4}\\ S_{3L,M5} \end{array}\right]$$

where S indicates an MID component of serine, the subscript m indicates a measured value, the subscript c indicates a value corrected for incorporation of naturally occurring isotopes, the subscript L (for *labeled*) preceded by a number indicates a molecule of serine guaranteed to have that number of labeled carbon atoms, and the subscript M followed by a number indicates the MID component corresponds to that number of atomic mass units above the monoisotopic mass of serine. As examples; $S_{m,M0}$ is the measured MID component of serine that is zero atomic mass units above the monoisotopic mass, $S_{1L,M2}$ is the theoretical MID component two atomic mass units above the monoisotopic mass of a serine molecule guaranteed to have one carbon derived from [U-^13^C_6_]glucose, and $S_{c,M1}$ is the corrected MID component of serine that is one atomic mass unit above the monoisotopic mass.

Equation (1) can be rewritten as Equation (2) because the multiplication of a matrix by a column vector is equivalent to weighting the columns of the matrix by the components of the vector [6].

(2) $$\left[\begin{array}{c} S_{m,M0}\\ S_{m,M1}\\ S_{m,M2}\\ S_{m,M3}\\ S_{m,M4}\\ S_{m,M5} \end{array}\right]=\left[\begin{array}{cccc} S_{0L,M0} & S_{1L,M0} & S_{2L,M0} & S_{3L,M0}\\ S_{0L,M1} & S_{1L,M1} & S_{2L,M1} & S_{3L,M1}\\ S_{0L,M2} & S_{1L,M2} & S_{2L,M2} & S_{3L,M2}\\ S_{0L,M3} & S_{1L,M3} & S_{2L,M3} & S_{3L,M3}\\ S_{0L,M4} & S_{1L,M4} & S_{2L,M4} & S_{3L,M4}\\ S_{0L,M5} & S_{1L,M5} & S_{2L,M5} & S_{3L,M5} \end{array}\right]*\left[\begin{array}{c} S_{c,M0}\\ S_{c,M1}\\ S_{c,M2}\\ S_{c,M3} \end{array}\right]$$

This elegant formulation, proposed by Brunengraber and colleagues [7], collects the corrected MID components as a vector and maps it to the measured MID by multiplication of what is termed a correction matrix. Equation (2) is written in succinct matrix form as Equation (3).

(3) $$\bar{S_{m}}=\bar{\bar{CM}}*\bar{S_{c}}$$

where $\bar{S_{m}}$ is the measured MID of serine, $\bar{\bar{CM}}$ is the correction matrix for serine in a ^13^C tracing experiment, and $\bar{S_{c}}$ is the MID of serine corrected for abundances of naturally occurring isotopes. Thus, the MID corrected for abundances of naturally occurring isotopes is given by Equation (4).

(4) $$\bar{S_{c}}={\bar{\bar{CM}}}^{+}*\bar{S_{m}}$$

where ${\bar{\bar{CM}}}^{+}$ is the Moore–Penrose inverse of the correction matrix. The above example is worked through for a ^13^C tracer and serine. However, it can be generalized to any tracer and metabolite by formation of analogous component vectors in Equation (1). PolyMID-Correct does not allow the corrected MID to have more components than physically possible, e.g., $\bar{S_{c}}$ can only have four components in a carbon-tracing experiment because serine only has three carbon atoms. However, $\bar{S_{m}}$ may have more than four components due to naturally occurring heavy isotopes of other atoms or those on derivatization reagents. This leads to situations where the correction matrix is not square and Equation (4) is overdetermined. Thus, Equation (4) yields a best fit for $\bar{S_{c}}$ with associated residuals. Application of this algorithm may also yield negative values for components of the corrected MID. Negative values do not have a physical significance and may arise due to imperfections in measurements. PolyMID-Correct reports these negative values as they are computed because replacing them with zeros would distort the results, e.g., the sum of the MID would no longer be one.

### 2.2. The Correction Matrix

As determined in the previous section, the component vectors of Equation (1) are the columns of the correction matrix. Herein, the algorithm applied to compute the columns of the correction matrix is presented. It is mathematically equivalent to a combinatorial probability strategy [8,9], though only columns corresponding to potentially labeled atoms of the measured metabolite are necessary to compute for its downstream application.

Each component vector in Equation (1) is an MID of serine, where serine is guaranteed to contain a specified number of ^13^C atoms from [U-^13^C_6_]glucose. The first component vector corresponds to serine with zero ^13^C atoms derived from the tracer, the second component vector corresponds to serine with one ^13^C atom derived from [U-^13^C_6_]glucose, etc. If the MID of an intact and unmodified serine has been measured, its chemical formula is: C_3_H_7_NO_3_. Each carbon on serine is capable of being labeled as ^13^C from the tracer. The first component vector has no ^13^C atoms derived from the tracer, so it is simply the MID of serine found in nature. The components of this vector can be computed from the isotope mass distributions (IMDs) of each atom [1], specified as Equation (5). Note the distinction between an IMD and an MID as illustrated in Figure 1A.

(5) $$\begin{array}{c} IMD_{C}=\left[\begin{array}{ccc} C_{M0} & C_{M1} & C_{M2} \end{array}\right]\\ IMD_{H}=\left[\begin{array}{ccc} H_{M0} & H_{M1} & H_{M2} \end{array}\right]\\ IMD_{N}=\left[\begin{array}{ccc} N_{M0} & N_{M1} & N_{M2} \end{array}\right]\\ IMD_{O}=\left[\begin{array}{ccc} O_{M0} & O_{M1} & O_{M2} \end{array}\right] \end{array}$$

where C, H, N, and O represent the elements for which they are chemical symbols. The subscript M followed by a number indicates the relative abundance of an isotope that is that number of atomic mass units heavier than the nominal mass. These values are known constants. To compute the MID of serine (C_3_H_7_NO_3_), the IMDs of the atoms are multiplied together as if they were polynomials [10] as in Equation (6).

(6) $$\begin{array}{l} MID_{C3H7NO3}=IMD_{C}{}^{3}*IMD_{H}{}^{7}*IMD_{N}*IMD_{O}{}^{3}\\ MID_{C3H7NO3}={\left[\begin{array}{ccc} C_{M0} & C_{M1} & C_{M2} \end{array}\right]}^{3}{\left[\begin{array}{ccc} H_{M0} & H_{M1} & H_{M2} \end{array}\right]}^{7}\left[\begin{array}{ccc} N_{M0} & N_{M1} & N_{M2} \end{array}\right]{\left[\begin{array}{ccc} O_{M0} & O_{M1} & O_{M2} \end{array}\right]}^{3} \end{array}$$

Terms that have the same collective mass are grouped as illustrated for a simple example molecule, C_2_, in Equation (7).

(7) $$\begin{array}{l} MID_{C2}=IMD_{C}{}^{2}\\ MID_{C2}=\left[\begin{array}{ccc} C_{M0} & C_{M1} & C_{M2} \end{array}\right]*\left[\begin{array}{ccc} C_{M0} & C_{M1} & C_{M2} \end{array}\right]\\ MID_{C2}=\left[\begin{array}{ccccc} C_{M0}C_{M0} & 2C_{M0}C_{M1} & \left(2C_{M0}C_{M2}+C_{M1}C_{M1}\right) & 2C_{M1}C_{M2} & C_{M2}C_{M2} \end{array}\right] \end{array}$$

The second column of the correction matrix in Equation (2) is the MID of a serine molecule as it is found in nature, but with one carbon replaced by a labeled carbon atom derived from the tracer, C_L_. Thus, the second column of the correction matrix is the MID of the molecule C_L_C_2_H_7_NO_3,_ where the IMD of C_L_ is considered to be 100% enriched for isotopes one atomic mass unit above the nominal mass, as per the definition of a labeled carbon atom.

(8) $$\begin{array}{l} \;\;IMD_{C_{L}}=\left[\begin{array}{ccc} C_{L,M0} & C_{L,M1} & C_{L,M2} \end{array}\right]\\ \;\;IMD_{C_{L}}=\left[\begin{array}{ccc} 0 & 1 & 0 \end{array}\right] \end{array}$$

where $IMD_{C_{L}}$ is the isotope mass distribution of a labeled carbon atom and $C_{L,M0}$ is the component of $IMD_{C_{L}}$ corresponding to 0 atomic mass units above the nominal mass of carbon. The number of atomic mass units above nominal mass is indicated by the number following M in the subscript.

Values within the remaining columns of the correction matrix are computed analogously, thus providing all information required to apply Equation (4) to correct for incorporation of naturally occurring heavy isotopes in a serine MID as measured in a ^13^C tracing experiment. This algorithm can be generalized to compute the correction matrix corresponding to any metabolite-tracer combination. For larger metabolites with tens of atoms that can acquire label from the tracer (e.g., lipids in a carbon tracing experiment), many expansions of long polynomials must be performed to compute the columns of the correction matrix; thus PolyMID-Correct may require more time to compute their corrected MIDs.

### 2.3. High Resolution Data

**Figure 2:**
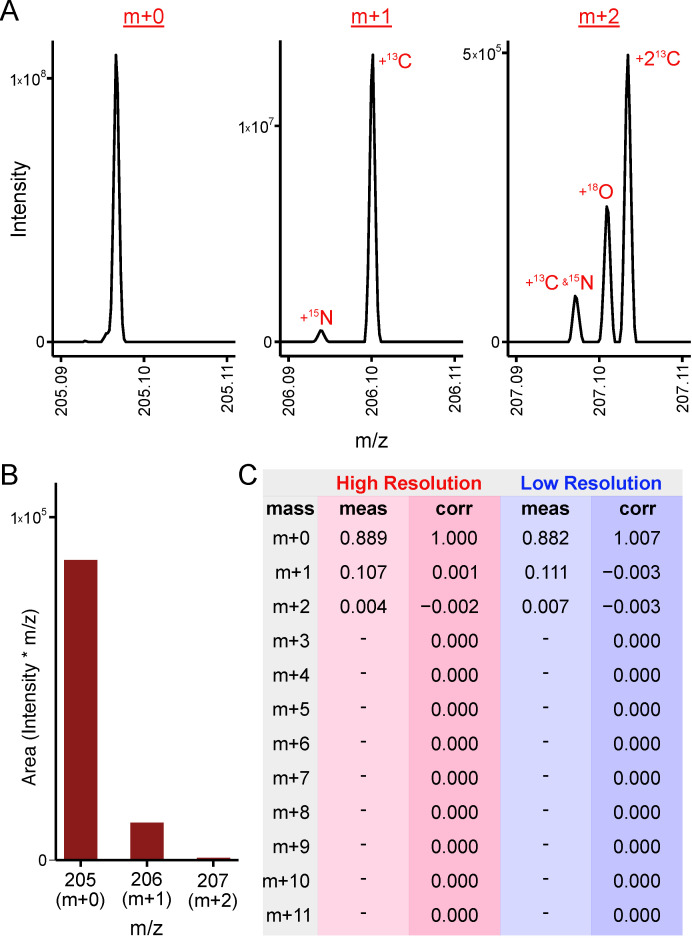
Correction of High- and Low- Resolution Data. (**A**) High-resolution mass spectrograph of tryptophan. (**B**) Low-resolution representation of the same mass spectrograph of tryptophan. (**C**) PolyMID correction of tryptophan MIDs for naturally occurring heavy isotope abundances in a ^13^C tracer experiment. Abbreviations: meas: measured, corr: corrected.

The distinction between high- and low- resolution mass spectrometer data in stable-isotope tracing is whether the mass differences due to incorporation of heavy isotopes of the tracer element can be distinguished from incorporation of those of other elements. This is illustrated in the mass spectrographs displayed in Figure 2A,B for a molecule of tryptophan. The spectrograph in Figure 2A is collected from a high-resolution Orbitrap instrument capable of resolving mass differences due to incorporation of heavy isotopes of carbon, nitrogen, oxygen, and hydrogen. The data in Figure 2B is a representation of the same data as it would be measured on a low-resolution instrument, i.e., one that is not capable of resolving mass differences due to incorporation of a heavy isotope of carbon from that of any other heavy isotope. PolyMID-Correct considers high resolution measurements by setting the IMD for elements whose isotope mass differences are resolved from those of the tracer element to be 100% nominal mass. The measured and corrected high- and low- resolution MIDs of tryptophan are displayed in Figure 2C. The m + 0 term (i.e., the term corresponding to zero atomic mass units above monoisotopic mass) is near unity when the measured MID of tryptophan is corrected for abundances of naturally occurring heavy isotopes because it was extracted from a sample not exposed to a stable-isotope tracer. High-resolution data is entered as the relative areas of peaks due to incorporation of heavy isotopes of atoms of the same element as the tracer. Thus, for tryptophan in a carbon tracing experiment, the m + 0 term would be the relative peak area corresponding to monoisotopic tryptophan (with no heavy isotopes of any atoms), the m + 1 term would be the relative peak area corresponding to tryptophan with one ^13^C atom, the m + 2 term would be the relative peak area corresponding to tryptophan with two ^13^C atoms, etc. Peak areas would be relative to the sum of the m + 0 peak and all peaks of tryptophan corresponding to incorporation of one or more ^13^C atoms.

### 2.4. Tracer Purity and Tracer Atom Enrichment

**Figure 3:**
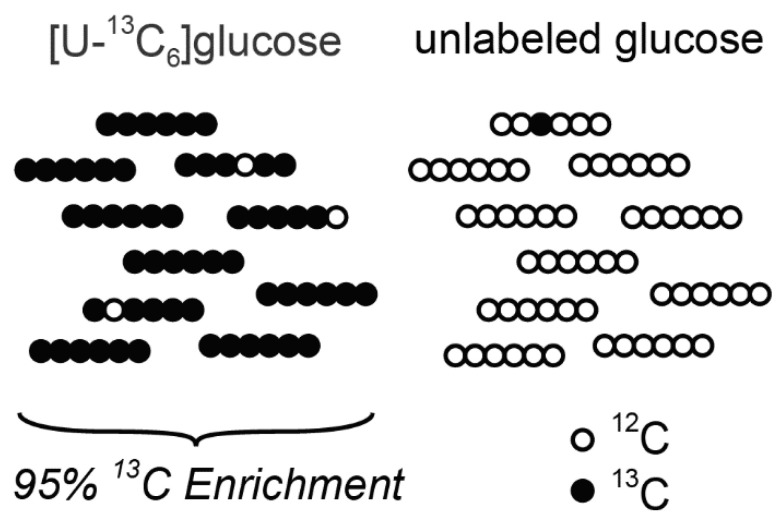
Illustrations of Tracer Enrichment and Label Enrichment. [U-^13^C_6_]glucose tracer enrichment is 50% and ^13^C label enrichment is 95%.

Stable-isotope tracing experiments may be performed with tracers that are less than 100% pure and/or having heavy isotope enrichments of less than 100% at the labeled atom positions (Figure 3). Consider an experiment performed with [U-^13^C_6_]glucose where it is 50% pure with an atom enrichment of 95%. After correcting the MID of serine for natural isotopic abundances, the labeling on serine can be interpreted as carbon atoms derived from labeled atoms of the tracer, i.e., 50% [U-^13^C_6_]glucose with 95% atom enrichment. However, after subsequent correction for tracer purity and enrichment, the labeling on serine can be interpreted as carbon atoms derived from glucose; a much more physiologically relevant readout. PolyMID-Correct removes the influences of tracer impurity and less than unity atom enrichment by adjusting the IMD of the labeled element as in Equation (9).

(9) $$IMD_{E_{L}}=P*E*\left[\begin{array}{ccc} 0 & 1 & 0\;\ldots \end{array}\right]+\left(1-P*E\right)*IMD_{E}$$

where P is the purity of the tracer, E is the labeled-atom enrichment of the tracer, $IMD_{E_{L}}$ is the isotope mass distribution of the labeled element, and $IMD_{E}$ is the isotope mass distribution of the element as it is found in nature. In other words, the IMD of the labeled element has a component that is 100% labeled and a component with that element’s naturally occurring IMD.

## 3. Methods

### 3.1. PolyMID Availability and Installation

PolyMID is free open source software available under the MIT license. Source code is available at http://VacantiLab.github.com/PolyMID (accessed on 10 May 2021). PolyMID runs on Python 3 and is operating system independent. Python 3 is required to install and run PolyMID. To determine if Python 3 is installed, type the following command in the Terminal (MacOS and Linux) or Cmd (Windows) window:

python3 --version

If a version of Python 3 is listed, then Python 3 is installed. If not, then Python 3 must be installed prior to continuing. With Python 3 installed, use the following command in the Terminal or Cmd window to install PolyMID:

pip install PolyMID

### 3.2. Running PolyMID-Correct

#### 3.2.1. Inputs from a Text File

The following provides instructions to perform the correction of a measured MID of tryptophan (Figure 2) for naturally occurring heavy isotope abundances. In a plain text editor, create a .txt file having the following format:

FragmentName: TryptophanProtonated

FragmentFormula: C11H13N2O2

CanAcquireLabel: C11H13N2O2

MIDm: 0.88885 0.106829 0.004322

LabeledElement: C

TracerEnrichment: 1

LabelEnrichment: 1

HighRes: N O H

*FragmentName* and *FragmentFormula* specify the name and formula of the metabolite fragment. For analyses on LCMS systems using electrospray ionization, the fragment will generally be the whole metabolite. For analyses on GCMS systems using electron ionization, the fragment will generally be a portion of the derivatized form of the whole metabolite. The value, *CanAcquireLabel*, specifies which atoms of the fragment can possibly acquire label from the tracer. Only the atoms that are of the same element as the tracer are considered. If the fragment formula is identical to the metabolite formula, then this input could be the same as the input *FragmentFormula*. However, in cases where the fragment is a derivatized version of the metabolite, atoms of the derivatizing agent cannot acquire label from the tracer and should be excluded from this input. The value, *MIDm*, is the mass isotopologue distribution of the metabolite fragment as it is measured. The value, *LabeledElement*, is the chemical symbol of the heavy isotope-labeled element on the tracer molecule. The value, *TracerEnrichment*, is the percent of the chemical species of the tracer molecule that is labeled. The value, *LabelEnrichment*, is the fraction of atoms in labeled positions on the tracer that are labeled as heavy isotopes. Finally, *HighRes* is a series of element chemical symbols, separated by spaces, whose mass shifts due to incorporation of heavy isotopes are distinguished from those of the labeled element. *HighRes* can also take on values of *all* or *none* to indicate mass shifts due to incorporation of heavy isotopes of all or no chemical species, respectively, can be distinguished from those of the labeled element.

Open the Terminal (MacOS or Linux) or Cmd (Windows) window and type the command, *Python3*, to start the Python3 interpreter. The Python3 interpreter should now be running in the Terminal or Cmd window. Type the command, *Import PolyMID*, to load the PolyMID software. PolyMID-Correct is part of the PolyMID software package. To run PolyMID-Correct, type the command, *Output = PolyMID.Correct()*. A window accessing the operating system’s directories will open. Navigate and select the text file specifying the input values as formatted according to the instructions above. The program will run and print the corrected MID and associated sum of squared residuals when finished. The corrected MID and sum of squared residuals can also be accessed with the commands *Output.MIDc* and *Output.SSE*.

#### 3.2.2. Inputs from the Command Line

The ability to define all inputs and call PolyMID-Correct directly from the command line allows it to be integrated into data processing workflows. The following provides instructions to perform the correction of a measured MID of tryptophan (Figure 2) for naturally occurring heavy isotope abundances. Open the Terminal (MacOS or Linux) or Cmd (Windows) window and type the command, *Python3*, to start the Python3 interpreter. The Python3 interpreter should now be running in the Terminal or Cmd window. Type the command, *Import PolyMID*, to load the PolyMID software. To implement the same correction for naturally occurring heavy isotopes as performed above, first define the following variables:

>>> FragmentName = 'TryptophanProtonated'

>>> FragmentFormula = 'C11H13N2O2'

>>> CanAcquireLabel = 'C11H13N2O2'

>>> MIDm = [0.88885, 0.106829, 0.004322]

>>> LabeledElement = 'C'

>>> TracerEnrichment = 1

>>> LabelEnrichment = 1

>>> HighRes = ['N', 'O', 'H']

Next, curate the variables into a single “Fragment” object, *Input*:

>>> Input = PolyMID.Fragment(FragmentName, FragmentFormula, CanAcquireLabel, MIDm, LabeledElement, TracerEnrichment, LabelEnrichmentHighRes)

Finally, run PolyMID-Correct passing it the *Input* variable:

>>> Output = PolyMID.Correct(Input)

The program will run and print the corrected MID and associated sum of squared residuals when finished. The corrected MID and sum of squared residuals can also be accessed with the commands *Output.MIDc* and *Output.SSE*.

## 4. Discussion

Interpretation of labeling patterns from stable-isotope tracing experiments is widely applied and dependent on accounting for the influence of naturally occurring heavy isotopes. Investigating flux through metabolic pathways is used to probe complex mechanisms in eukaryotic systems, including: compartmental regulation of cellular metabolism [11,12], stress-induced adaptations of tumor cells [13], nutrient selection for biosynthesis [14,15], and the maintenance of stem cell pluripotency [16,17]. It is also used in processes to optimize production of desirable molecules in micro-organisms [18] and to investigate the function of the human microbiome [19]. 

Furthermore, a push towards understanding biology on a genome-wide level lends towards combining systems analyses of metabolic function, e.g., stable-isotope tracing measurements, with high-throughput molecular measurements. Considering the proteome as a purveyor of gene function: the increased depth and sharpened quantitative resolution of proteomic methods allows tissue proteome profiles to be linked to distinct metabolic phenotypes [20], while the ever-increasing sensitivity of detecting protein phosphorylation provides troves of data linking cell signaling cascades to stresses impacting metabolism [21,22,23,24]. Thus, clean interpretations, less the influence of natural isotopic abundances, of label incorporation from stable-isotope tracing-based measurements will be indispensable when systems analyses of genome-wide molecular measurements [25] are applied to advance understanding of the interplay between gene function and nutrient metabolism [26,27]. PolyMID-Correct, with options for single text-file inputs or a full command-line interface, is well-suited to complement other systems measurements as stand-alone software, or as fully integrated into computational pipelines analyzing multiple levels of systems measurements.

## Data Availability

Not applicable.
